# Effectiveness of Yiqi Fumai lyophilized injection for acute heart failure: Rationale and design of the AUGUST-AHF cohort study

**DOI:** 10.3389/fcvm.2022.1074406

**Published:** 2023-01-10

**Authors:** Xuecheng Zhang, Jing Kang, Jingjing Zhang, Ying Chen, Hengheng Dai, Mingzhi Hu, Yan Liu, Hongcai Shang

**Affiliations:** ^1^Key Laboratory of Chinese Internal Medicine of Ministry of Education, Dongzhimen Hospital, Beijing University of Chinese Medicine, Beijing, China; ^2^College of Traditional Chinese Medicine and College of Integrated Chinese and Western Medicine, Nanjing University of Chinese Medicine, Nanjing, China; ^3^School of Public Health, Department of Global Health, Peking University, Beijing, China; ^4^Department of Traditional Chinese Medicine, The Sixth Medical Center of the General Hospital of the Chinese People’s Liberation Army, Beijing, China

**Keywords:** Chinese medicine, Chinese herbal injections, acute heart failure, cohort study, real-word evidence

## Abstract

**Introduction:**

The effect of Yiqi Fumai lyophilized injection (YQFM) on acute heart failure (AHF) patients has been evaluated in a large sample, randomized, controlled trial (AUGUST-AHF RCT study). However, restrictive eligibility criteria from a randomized clinical trial may raise concerns about the generalizability of the results to under-represented groups or complex patients with multimorbidity. Therefore, we intend to conduct the AUGUST-AHF cohort study which aims to assess the effectiveness of YQFM in patients with AHF in a real-world setting and compare the results with AUGUST-AHF RCT study.

**Methods and analysis:**

This prospective, multicenter cohort study will be conducted at 50 secondary and tertiary hospitals in China and comprise 1,200 patients with AHF. The participants will be followed for up to at least 180 days. The primary outcome is a composite of 90-day all-cause mortality or readmission for heart failure. The secondary outcomes include length of hospital stay, cardiac-specific death, MACE, NYHA cardiac function classification. Cox proportional-hazards regression models will be used to estimate the association between YQFM use and the primary outcome. The primary analysis will use propensity-score matching methods to balance the differences in baseline variables between treatment cohorts.

**Ethics and dissemination:**

Approval for the study has been obtained from the Ethical Committee of Dongzhimen Hospital (approval No. 2022DZMEC-327-02) and registered at ClinicalTrials.gov (NCT05586048). The study results will be published in peer-reviewed journals and presented at scientific conferences.

## 1. Introduction

Acute heart failure (AHF) occurs when the systemic circulatory system fails due to structural or functional heart abnormalities that are severe enough to necessitate hospitalization or emergency department visits (1). Once hospitalized, about 45% of AHF patients will be re-hospitalized or die within one year, imposing a heavy burden on patients and national healthcare systems worldwide (2, 3). During hospitalization, the pharmacological treatment for AHF consists primarily of intravenous diuretics with adjunctive vasodilators, inotropes, or vasopressors, which has minimal efficacy in improving post-discharge outcomes (1, 4). SGLT2 inhibitor empagliflozin has been preliminarily proven to have a long-term mortality benefit on AHF patients in recent months (5); nevertheless, their use remains limited in clinical practice due to cost, side-effect, and lack of availability in local pharmacies (6, 7).

In China, traditional Chinese medicine (TCM) has been widely used in the treatment of heart disease for thousands of years. Yiqi Fumai lyophilized injection (YQFM) is an injection from Chinese medicine extraction processed by modern technology and is derived from the ancient formula Shengmai powder composed of Panax ginseng, Ophiopogon japonicus, and Schisandra chinensis (8). It has been applied clinically to treat heart failure (HF) as an important supplementary drug and is included in the latest Chinese Health Insurance Catalog (9). YQFM significantly improves cardiac function and related indicators and relieves cardiac load and related symptoms among HF patients (10). Pharmacological studies have demonstrated that YQFM can inhibit myocardial remodeling, attenuate cardiomyocyte hypertrophy and apoptosis, and improve the cardiomyocytes’ energy metabolism, which is achieved by various signaling pathways (11).

We are conducting a large sample, randomized, double-blind, placebo-controlled trial (AUGUST-AHF RCT study) to evaluate the YQFM effect on the 90-day mortality or readmission rate in AHF patients (8). However, restrictive eligibility criteria from a randomized clinical trial (RCT) may raise concerns about the generalizability of the results to under-represented groups or complex patients with multimorbidity, as seen in routine clinical practice (12). A tight regulatory framework may be appropriate for early-phase RCT, but its ubiquitous application to clinical trials may stifle the ability to test the YQFM. In some published studies, RCT results are inconsistent when the treatment or drugs are transferred to clinical practice (13, 14). Thus, observational data from a real-world study may be needed to address some AUGUST-AHF RCT study limitations, providing an effectiveness evaluation applicable to a broad population in routine clinical practice.

**FIGURE 1:**
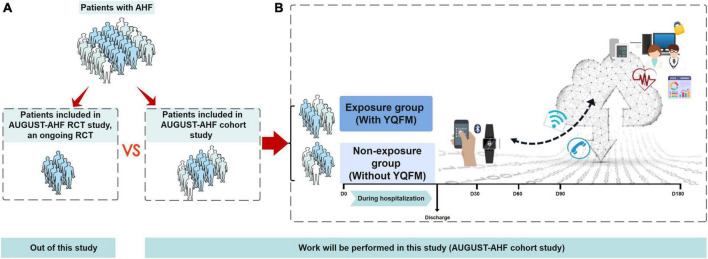
Overview of study objectives and flow diagram of AUGUST-AHF cohort study. **(A)** Out of this study. Of the total population of AHF people with yiqi fumai lyophilized injection (YQFM), only a subset are included in randomized controlled trials (RCT), based on the RCT inclusion/exclusion criteria. For this study, the specific RCT of interest is the AUGUST-AHF RCT study, an ongoing RCT which also performed by our team, evaluating the efficacy of YQFM on long-term prognosis. The RCT generates results that inform clinical practice, and the anonymized raw data for the study will be made available to us. **(B)** Work will be performed in this study (AUGUST-AHF cohort study). This cohort study will comprise patients with AHF, and they will be divided into unexposed and exposed groups according to whether are taking YQFM in real clinical practice. The primary outcome will be a composite of 90-day all-cause mortality or readmission for heart failure, which is the same as AUGUST-AHF RCT study. During 180- day follow-up, we will provide patients with smart watches to monitor their body weight, blood pressure, and heart rate. Smartphone-based clinical reporting applications will also be used to manage patients. Objective 1: thorough our cohort study, assess the effectiveness of YQFM on the 90-day mortality or readmission rate in patients with AHF in real-world setting. Objective 2 compare the result of our study with AUGUST-AHF RCT study. Objective 3 determine the representativeness of the AUGUST-AHF RCT study populations to real-world patients, and describe differences in the characteristics of patients who met the RCT eligibility and those not in AUGUST-AHF cohort study. AHF, acute heart failure; YQFM, yiqi fumai lyophilized injection; RCT, randomized controlled trial.

Here, we intend to conduct the AUGUST-AHF cohort study (Figure 1), which aims to (1) assess the effectiveness of YQFM on the 90-day mortality or readmission rate in patients with AHF in a real-world setting; (2) compare the results of our study with AUGUST-AHF RCT study; (3) determine the representativeness of the AUGUST-AHF RCT study populations to real-world patients, and describe differences in patients’ characteristics who met the RCT eligibility and those not in AUGUST-AHF cohort study.

## 2. Materials and methods

### 2.1. Study design

This prospective, multicenter cohort study will be conducted at 50 secondary and tertiary hospitals. Approval for the study has been obtained from the Ethical Committee of Dongzhimen Hospital (approval No. 2022DZMEC-327-02). The study has been registered at ClinicalTrials.gov (NCT05586048).

### 2.2. Eligibility criteria and recruitment

Patients diagnosed with AHF will be eligible candidates to provide more generalizable clinical presentations in the real world. Between December 2022 and November 2025, these study participants will be recruited from 50 centers. Patients will be referred by cardiologists in the cardiology ward or coronary care unit (CCU) and screened by professional examiners according to inclusion and exclusion criteria (details shown in Table 1, population of AUGUST-AHF cohort study). A research assistant will obtain written informed consent from the patients who meet the inclusion criteria.

**TABLE 1:** Comparison of AUGUST-AHF cohort study and AUGUST-AHF RCT study.

	AUGUST-AHF cohort study	AUGUST-AHF RCT study
Population	Inclusion criteria 1. Diagnosis of AHF 2. Age ≥ 18 years 3. Voluntarily participate in and sign the informed consent form; Exclusion criteria 1. With major psychiatric disorders or unable to complete follow-up assessment	Key inclusion criteria 1. Diagnosis of AHF 2. Age ≥ 18 years 3. Voluntarily participate in and sign the informed consent form; 4. Randomization will have to be completed within 16 h of presentation Key exclusion criteria 1. SPB ≥ 90 mmHg prior to enrolment or patients with uncontrolled hypertension 2. Severe bleeding or coagulation disorders 3. Major neurological events 4. Known liver damage or potentially severe liver disease 5. Severe renal insufficiency or planned or under dialysis 6. AHF due to significant arrhythmias 7. Known to have acute myocarditis, obstructive hypertrophic cardiomyopathy, complex congenital heart disease, constrictive or restrictive pericarditis, cardiac tamponade, severe aortic stenosis, severe mitral stenosis, severe aortic, or mitral regurgitation 8. Dyspnoea due to obvious non-cardiac causes 9. Patients who have received any organ transplant 10. Current mechanical ventilation or circulation support 11. Pregnant or lactating women 12. with major psychiatric disorders, a history of any organ malignancy, or Yiqi Fumai lyophilized injection allergy
Intervention/ Exposure and comparison	Exposed group: with Yiqi Fumai lyophilized injection during the hospitalization Non-exposed group: without YiqiFumai lyophilized injection during the hospitalization	Treatment group: Yiqi Fumai lyophilized injection + standardized western medications Control group: Placebo (5% glucose or 0.9% normal saline injection 250 mL) + standardized western medications
Outcome	Primary outcome: 90-day all-cause mortality or HF readmission Secondary outcomes: 180-day all-cause death or HF readmission, length of hospital stay, 90-day cardiac-specific death, MACE, dyspnoea via VAS, dyspnoea via Likert 7-point scale, NYHA function class, MLHFQ scale, MMAS-8, heart rate at follow-up	Primary outcome: 90-day all-cause mortality or HF readmission Secondary outcomes: 180-day all-cause mortality or HF readmission rate, length of hospital stay, 90-day cardiac-specific death, MACE, dyspnoea via VAS score, dyspnoea via Likert 7-point scale, NYHA functional class, WHF, MLHFQ scale, intravenous diuretics, NT-proBNP
Follow-up	180 days	180 days
Study design	Cohort study	RCT

HF, heart failure; MACE, major cardiovascular adverse event; VAS, dyspnoea via visual analog scale; MLHFQ, minnesota living with heart failure quality of life; MMAS-8, morisky medication adherence scale; RCT, randomized controlled trial.

### 2.3. Exposure

This is an observational study, and there will be no intervention during the diagnosis and treatment process. Respecting their individual preferences, all patients will receive conventional treatment during their hospitalization at their physicians’ discretion, including intravenous diuretics with adjunctive vasodilators, inotropes, and other therapies when necessary. Patients will be divided into exposed and non-exposed groups according to whether they receive YQFM treatment during the hospitalization. The average hospitalization duration for AHF patients is around 10 days in China (15), and prior studies have primarily adopted 10-day injections as a course of treatment (8). However, patients sometimes can’t complete the 10-day course of YQFM treatment in clinical practice, due to subjectivity, complexity, and adherence of patients and physician, or potential economic burden (16). Thus, we pre-defined half of the standard course of treatment as the boundary between low exposure and low exposure, based on clinical experts’ opinion. patients who receive YQFM for less than 5 days and 5 days or more during their hospital stay will be defined as having low and high exposure, respectively.

### 2.4. Outcomes

The primary outcome is a composite of 90-day all-cause mortality or readmission for HF. The secondary outcomes are 180-day all-cause death or HF readmission, cardiac-specific death, major cardiovascular adverse event (MACE), length of hospital stay, dyspnoea by visual analog scale (VAS), dyspnoea by Likert 7-point scale, New York heart association (NYHA) cardiac function classification, Minnesota Living with Heart Failure Quality of Life (MLHFQ) scale, and Morisky Medication Adherence Scale (MMAS)-8. The cardiac-specific death is defined as death due to myocardial infarction (MI), worsening heart failure, death due to cardiovascular procedures, or other cardiovascular causes. MACE is defined as any of the following: death, MI, stroke, hospitalization because of HF, and revascularization, which includes percutaneous coronary intervention and coronary artery bypass grafting.

### 2.5. Study assessment

Researchers will assess patients daily during their hospitalization until their discharge and at the follow-up (details shown in Table 2). After patients have been enrolled in this study, researchers will examine their eligibility for the AUGST-AHF RCT study. The use of YQFM will be evaluated based on the daily medication record. The MACE endpoints and cardiac-specific death will be assessed and determined by the endpoint committee. VAS and 7-point Likert scale will be used to assess dyspnea by patient self-report, and the two scales will be measured at approximately the same time in patients’ supine position to ensure consistency. The MLHFQ scale consists of 21 questions and has been widely used to assess HF patients’ life quality (17). The Chinese version of MMAS of eight items has been extensively validated in China (18). It shows reliability and validity for HF patients (19), which would be a suitable tool for our study to assess patients’ medication adherence. Adverse events (AEs) will be monitored until Day 10 or until patients are discharged and significant AEs until Day 180.

**TABLE 2:** The schedule of assessment and data collection.

	Baseline	During the patient’s hospitalization^1^										Follow-up			
**Time point**	**Day 0**	**Day 1**	**Day 2**	**Day 3**	**Day 4**	**Day 5**	**Day 6**	**Day 7**	**Day 8**	**Day 9**	**Day 10**	**Day 30**	**Day 60**	**Day 90**	**Day 180**
**Screening procedures**															
Eligibility screen	X														
Informed consort	X														
**Data collection and assessments**															
Basic information^2^	X														
AUGUST-AHF eligibility judgement^3^	X														
YQFM usage		X	X	X	X	X	X	X	X	X	X				
Physical examination*	X	X	X	X	X	X	X	X	X	X	X				
Weight*	X	X	X	X	X	X	X	X	X	X	X				
Total intravenous diuretics*	X	X	X	X	X	X	X	X	X	X	X				
Laboratory inspection*	X										X				
Myocardial injury biomarker*	X														
ECG*	X										X				
Echocardiogram*	X														
BNP or NT-proBNP*	X														
NYHA cardiac function classification	X										X			X	
Dyspnoea via VAS	X	X	X	X	X	X	X	X	X	X	X				
Dyspnoea via Likert 7-point comparator scale	X	X	X	X	X	X	X	X	X	X	X				
MLHFQ scale	X										X	X	X	X	X
Length of hospital and CCU stay											X				
Readmission for HF												X	X	X	X
All-cause death												X	X	X	X
Heart rate												X	X	X	X
MMAS-8												X	X	X	X
MACE												X	X	X	X
Adverse and serious adverse events	X	X	X	X	X	X	X	X	X	X	X	X	X	X	X
Medications^4^	X	X	X	X	X	X	X	X	X	X	X	X	X	X	X

^1^If the hospital stay is less than 10 days, the necessary assessment will be completed before discharge. If the patient stays for more than 10 days, the use of Yiqi Fumai will be completed daily in the remaining hospital stay, and the MLHFQ scale will be additionally completed at discharge. ^2^Basic information concludes patient’s demographic information, comorbidity, disease history, alcohol and tobacco history, allergy history, and influenza vaccination history. ^3^AUGUST-AHF eligibility determination: The patient will be evaluated by the researchers according to the eligibility of the AUGST-AHF to determine whether the patient met the criteria. ^4^Medications will be recorded at baseline, during hospitalization, and follow-up period, especially drugs with clear beneficial effects in patients with heart failure, such as SGLT2, ARNI, ACEI, ARB, and β-blocker. *Non-required items, which will be obtained from electronic medical record if recorded. YQFM, Yiqi Fumai lyophilized injection; ECG, electrocardiogram; NYHA, New York heart association; MLHFQ, minnesota living with heart failure quality of life; CCU, coronary care unit; MMAS, morisky medication adherence scale; MACE, major cardiovascular adverse event; SGLT2, sodium-glucose co-transporter 2; ARNI, angiotensin receptor-neprilysin inhibitor; ACEI, angiotensin-converting enzyme inhibitors; ARB, angiotensin receptor blockers.

### 2.6. Data collection and management

#### 2.6.1. Data collection during hospitalization

Identities of patients will be encoded with numbers to protect their privacy. At enrolment, patients’ basic information will be obtained, including demographic information, disease history, alcohol, cigarette use, allergy history, and influenza vaccine history. Moreover, information on laboratory examination, imaging examination, and medications will be collected. The laboratory inspection includes biochemistry, hematology, and routine urine analysis. The medication information, including the name, the dosage, and the duration of the medication use, will be recorded throughout the study. More details about data collection across time points are shown in Table 2.

#### 2.6.2. Follow-up

Following enrolment, patients will be monitored for a minimum of 180 days, with visits occurring at 30, 60, 90, and 180 days. During follow-up, we will use a smartphone-based clinical reporting application (app) to manage patients. Patients will fill out the MLHFQ and MMAS-8 scales online by this app. Before discharge, the doctor will explain the details of the app’s scales to patients and their relatives until they fully understand. Concurrently, the app will display essential discharge instructions and details. Within seven days of each visit time point (30, 60, 90, and 180 days), an independent investigator will confirm survival status, HF readmission, NYHA cardiac function classification, MACE, and AEs of patients by telephone or outpatient, and remind patients to complete the app questionnaire in the app. We will provide patients with smartwatches and ask them to wear these smartwatches during follow-up, which could monitor their body weight, BP, and heart rate. The records will be automatically uploaded to the app through Bluetooth. All data will be sent over the internet to a back-end infrastructure on secure servers. Moreover, this specially designed app gives researchers secure online access to scale information and patients’ vital-sign readings.

#### 2.6.3. Data management

All data will be collected by Case Report Form (CRF) of a paper version and then transcribed to electronic data capture (EDC) system. To ensure data accuracy, data entry personnel will receive the necessary training and perform entry while checking for any suspicious parameters. The data will be monitored regularly for quality control by a data management team. Team members will periodically check the validity and logicality of the data through the EDC system and send electronic queries to the fillers in case of any questions until the data is confirmed to be correct. The team will also organize regular visits to centers and check the paper consistency and electronic CRFs.

### 2.7. Sample size

Based on the results of previous observational studies, the composite outcome of 90-day all-cause mortality or readmission for HF was approximately 25% among patients who received conventional therapy for acute heart failure (20, 21). We assumed that the composite rate of those who combined with YQFM in clinical practice is 18%. Setting a significance level of two-sided alpha level of 0.05 and 10% of the loss to follow-up, 1,200 patients would be enough to detect the composite outcome with 80% power.

### 2.8. Statistical analysis

The baseline characteristic and observational data will be expressed as means ± standard deviation, medians, or percentages, and performed an independent t-test, Mann-Whitney U test, or Chi-square test/Fisher’s exact test, depending on the distribution.

Cox proportional-hazards regression models will be used to estimate the association between YQFM use and the primary outcome. An initial multivariable Cox regression model will include demographic factors, clinical factors, laboratory tests, and medications.

The primary analysis will use propensity-score matching methods to balance the differences in baseline variables between treatment cohorts. The individual propensities for receipt of YQFM treatment will be estimated with the use of a multivariable logistic regression model that include the same covariates as the Cox regression model. In the propensity-score matching analysis, the nearest-neighbor method will be applied to create a matched control sample. All variables will include in the propensity score model reflected knowledge available at baseline. Missing baseline variables will be handled by multiple imputation by chained equations using the other variables available. To verify the covariate balancing after the propensity score matching, the standardized mean difference (SMD) will be calculated with a threshold of 10% designated to indicate clinically meaningful imbalance (22). Kaplan-Meier curves and Cox models that use the propensity-score matching will be reported. A sensitivity analysis for unobserved confounding will be performed by using the Rosenbaum’s method to assess the robustness of findings due to hidden bias (23).

We will compare our findings with the AUGUST-AHF RCT study to determine whether the results, particularly the primary outcome, are compatible between the two. The proportion of patients who met the eligibility requirements for the AUGUST-AHF RCT study will be computed. Differences in baseline characteristics between those who met the RCT eligibility and those who did not will also be tested by the statistical methods described above.

We will perform an interim analysis when 33% of total patients (N = 400) have completed the 90-day follow-up. The interim analysis will be performed by independent statisticians, and we may adjust the course of the subsequent study or revise the study protocol based on the analysis results.

All statistical analyses were performed with the R statistical package version 4.0.2 (R Foundation for Statistical Computing, https://www.R-project.org/). Furthermore, two-tailed p < 0.05 was considered statistically significant.

### 2.9. Ethics and dissemination

The study has been approved by the Ethical Committee of Dongzhimen Hospital, and ethical approval will be obtained from the local ethics committees of the other centers. The amended version of the protocol will then be published to the Chinese Clinical Trial Registry after receiving ethical approval for any modifications. The study will be conducted per the Helsinki declaration, in which patients must provide written informed consent before participation. The study results will be published in national and international peer-reviewed journals and presented at scientific conferences. We plan to communicate our findings to potential or diagnosed AHF patients and their families to help them understand the risks and benefits of drug use.

## 3. Discussion

This is the first large prospective cohort study to evaluate the Chinese herbal injection effectiveness in AHF patients. This Chinese herbal injection, YQFM, is being studied in large RCTs such as AUGUST-AHF and applied clinically to treat AHF. Although the data from RCTs could provide critical evidence of clinical activity, the strict eligibility criteria may raise concerns about generalizability. Therefore, it is essential to evaluate the effectiveness of YQFM in a real-world study.

### 3.1. Drug rationale

Yiqi Fumai lyophilized injection is accepted by most patients in China because of its effectiveness and safety, and can be used as an important supplementary drug for HF treatment. At a pathophysiological level, YQFM improves coronary blood flow, reduces myocardial oxygen consumption, inhibits inflammatory mediators, and reduces myocardial injury (24). The potential mechanism may be multifactorial. At a molecular level, it could improve cardiac tissue damage and fibrosis and reduce the serum levels of BNP, CK, LDH, and hs-CRP (25, 26). It has also been discovered to improve the mitochondrial function of cardiomyocytes by regulating mitochondrial biogenesis-related proteins and inhibiting apoptosis while attenuating cardiac hypertrophy by autophagy regulation through the PI (3) K/AKT/mTOR pathway (11, 27).

Furthermore, YQMF attenuates doxorubicin-induced cardiotoxicity, hepatotoxicity, and nephrotoxicity in rats by oxidative stress inhibition, inflammation, and apoptosis (28). In a meta-analysis of 33 RCTs (10), YQFM combined with standard HF therapy was reported to have a significantly higher likelihood of improving treatment response. According to reports, YQFM considerably improved heart function, the performance of the 6-Minute Walk Test, and the patient’s quality of life, as well as lowered NT-proBNP levels. The AEs and clinical safety of YQFM has been well documented and assessed in previous studies. Potential adverse effects of YQFM mainly include pruritus, rashes, nausea, palpitations, and drowsiness, and most of them were remitted after treatment (10, 29). Safety surveillance studies with large sample sizes have been performed, which reported low incidence of AEs from 0.176 to 1.25% (29–31). Several systematic reviews of RCTs also reported that an acceptable safety profile (10, 32, 33). Overall, YQFM has been demonstrated to be safe, with very low incidence of AEs.

### 3.2. Exposure rationale

#### 3.2.1. Endpoints and outcome measures rationale

Since patients are prone to recurrence with exacerbation and have a high risk of death and readmission during the vulnerable 90 days after HF hospitalization (34, 35), we focused on this 90-day composite endpoint, a high-risk period for AHF patients that require special attention and close follow-up (36, 37). Besides, evidence has shown that HF patients’ vital signs (e.g., heart rate) and medication adherence after discharge are prognostic factors (38–40). However, many efficacy studies of pharmacological interventions have neglected this association and have not collected this data. Therefore, we used the smartwatch and app to collect information on vital signs and medication adherence during follow-up. Meanwhile, patients might be remotely monitored through the app to save time and money on unnecessary travel. The use of smartwatches could better monitor patients’ health conditions. Innovative models of remote monitoring based on technological advances may help overcome the challenges of long follow-up management for patients and reduce shedding rates.

#### 3.2.2. Study design rationale

In our study, we will apply a broader eligibility criterion and nearly the same outcome measures as the AUGUST-AHF RCT study (shown in Tables 1, 2). The study results will represent the long-term effectiveness of YQFM in a real-world setting.

Furthermore, we will compare the population and results of our study with the AUGUST-AHF RCT study. Complex patients with multimorbidity or a history of specific treatments, like heart transplantation, will be excluded from the AUGUST-AHF RCT investigation, raising concerns regarding the trial’s generalizability and the unknown benefit and risk of Yiqi Fumai Lyophilized Injection for this group. In our study, we specifically set up an assessment to determine whether patients are eligible for the AUGST-AHF RCT study. By comparing the populations of the two studies, we could determine the representativeness of the AUGUST-AHF RCT study populations to real-world patients and describe differences in the characteristics of patients who met the RCT eligibility criteria and those who did not participate in a real-world setting, something that is rarely mentioned in existing studies. Those who do not meet the AUGUST-AHF eligibility criteria will be analyzed separately to obtain efficacy in an underrepresented population.

Some studies have demonstrated that the outcomes of patients in clinical trials differ from those in real-world settings, with lower survival rates and a higher incidence of AEs (13, 14). The results of our study will be compared with AUGUST-AHF to verify whether this condition also exists in HF patients and to analyze its possible causes. The outcome measures are largely the same between the two studies, facilitating data comparability. Furthermore, differences in compliance between trial and non-trial patients have been cited as an important reason for differences in results (39). Thus, we will include compliance analysis as an important part of the study to explore the differences in compliance between patients in and out of clinical trials and their impact on clinical outcomes. The study outcomes will aid patients, physicians, and policymakers in selecting the optimal treatment options for all patient subgroups. The method could potentially serve as a model for utilizing RCT-validated data in routine clinical practice.

In this cohort study, it was nearly impossible to remove biases. First, participating centers are all tertiary hospitals. Thus, referral bias could be present. Considering AHF as a serious to life-threatening acute disease, patients with suspected AHF are almost exclusively referred to tertiary hospitals in China, and few patients are left in community hospitals. This may weaken the impact of referral bias. Moreover, propensity score methods will balance the two cohorts’ baseline characteristics, avoiding selection bias. Second, the study will involve numerous patient assessments, and both the proficiency of the assessors and the way of questioning may affect the assessment’s reliability, leading to measurement bias. Therefore, we will conduct uniform training for the assessors to avoid it. Third, telemedicine and telemonitoring (the smartwatch and app) may reduce mortality and readmission, resulting in confounding bias. However, in this study, both the exposed and the non-exposed group will be set up with the same telemedicine and telemonitoring, which may eliminate the influence of this confounding factor.

## Ethics statement

The studies involving human participants were reviewed and approved by Ethical Committee of Dongzhimen Hospital. The patients/participants provided their written informed consent to participate in this study.

## Author contributions

YL and XZ conceived the study. HS provided practical suggestions on the design of the study. YL gave statistics related suggestions. XZ drafted the manuscript, and all the authors critically revised it. All authors contributed to the article and approved the submitted version.
